# Supplementary material for: Behavioral problems in anxious youth: Cross-sectional and prospective associations with reinforcement sensitivity and parental rejection
Source: PLoS One. 2022 Oct 27;17(10):e0267177. doi: 10.1371/journal.pone.0267177 (PMC9612547; doi:10.1371/journal.pone.0267177)
Supplement: S1 Fig — (DOCX) [file pone.0267177.s001.docx]

| 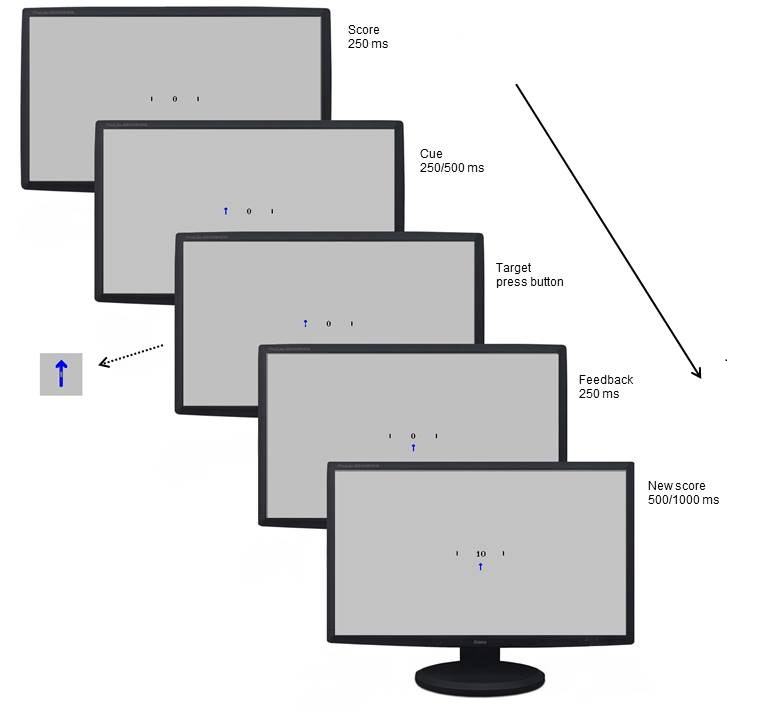 |
| --- |
| *Figure S1.* Example of a blue cue, cued trial with a sufficiently fast response in a winning game. This figure was previously published in: “Jonker, N. C., Glashouwer, K. A., Hoekzema, A., Ostafin, B. D., & de Jong, P. J. (2020). Heigtened self-reported punishment sensitivity, but no differential attention to cues signaling punishment or reward in anorexia nervosa. *PLOS ONE. https://doi.org/10.1371/journal.pone.0229742* |
