# Supplementary material for: Behavioral problems in anxious youth: Cross-sectional and prospective associations with reinforcement sensitivity and parental rejection
Source: PLoS One. 2022 Oct 27;17(10):e0267177. doi: 10.1371/journal.pone.0267177 (PMC9612547; doi:10.1371/journal.pone.0267177)
Supplement: Attachment — Submitted filename: rebuttal.docx [file pone.0267177.s002.docx]

| Dear Dr. Carels,  We would like to thank you for the invitation to revise and resubmit our manuscript – PONE-D-21-09707- entitled: “Behavioral Problems in Anxious Youth: Cross-Sectional and Prospective Associations with Reinforcement Sensitivity and Parental Rejection”.  We highly appreciate the constructive and helpful comments of the reviewers. In line with the suggestions, we carefully revised the manuscript. We now clearly indicate the added value of our research to previous conducted research and conducted the suggested moderation analysis. On the following pages, we have detailed how we addressed the comments and suggestions.  We hope that you will consider the comments to be adequately addressed and find the revised manuscript suitable for publication in PLOS ONE.  With warm regards | |
| --- | --- |
| Reviewer #1: The present study examined potential risk factors (e.g., punishment (PS) and reward sensitivity (RS) and parental rejection) for comorbid anxiety and behavioural problems in adolescents. PS/RS were not found to be associated with co-occurring behavioural problems in anxious youth either cross sectionally or prospectively. Behavioural problems predicted anxiety in young adulthood after controlling for adolescent anxiety. Contrary to expected this relationship was not mediated by parental rejection. |  |
| This paper has a number of strengths including it longitudinal design and the inclusion of multi method assessment (e.g., self report and behavioural).The study is likely to be of interest to readers of the journal and contributes to our understanding of co-occurring anxiety and behavioural problems which is important given that young people who present with this comorbidity often have a poorer prognosis and response to treatment. Weaknesses of the study include the use of an attentional punishment and reward sensitivity measure only and a lack of a use of diagnostic interviews to assess anxiety and behavioural problems. A few minor comments for discussion below | We thank the reviewer for his/her helpful comments. We adjusted our manuscript in line with the comments of the reviewer by highlighting how the current study is distinct from the other TRAILS study, by including a figure of the SOT and by adjusting the typo in table 1b. |
| I was surprised to see the authors introduce another TRAILS study (Kreuze, Jonker et al., 2020) similarly examining the relationships between punishment sensitivity and reward sensitivity anxiety/behavioural problems in the larger cohort in the discussion on the final page. It is suggested that the authors introduce this study in the introduction and clearly highlight how the present subsequent study is distinct from it and extends upon it to answer a new research question. | In line with the reviewer’s suggestion to introduce this study in the introduction, we now clearly highlight how the current study is distinct from the previous study and extends upon it, in the current paper as follows (page 4-5):  “In the entire focus group (*Ncross-sectional =* 696, *Nlongitudinal* = 598)*)* of the TRAILS cohort, we failed to find meaningful cross-sectional and prospective associations between punishment sensitivity (as indexed with the SOT) and anxiety symptoms and between reward sensitivity (as indexed with the SOT) and behavioral problems in CYP (31). The non-significant association between reward sensitivity and behavioral problems in this entire focus group may be explained by previous research that found that for non-clinical CYP, reward sensitivity may be actually associated with positive social and environmental functioning (26). The association between reward sensitivity with negative emotions and behavioral problems may especially hold for a clinical group. Also, the predicted association between punishment sensitivity and behavioral problems was not tested in this previous study. This pattern of punishment sensitivity leading to anxiety and behavioral problems may be especially relevant for highly anxious CYP. Especially these highly anxious CYP may react with anger/oppositional behaviors to escape from threatening situations, whereas this is not the case for low anxious children. Therefore, our current predictions specifically apply to a highly anxious sample, as was used in the current study. |
| To assist with explaining the SOT is it at all possible to include some sort of diagram. | In order to assist in explaining the SOT we included the figure S1 of the SOT in the supplement and refer to this in our method section (page 13) as follows:  “Figure S1 in the supplement shows the critical elements of the SOT.” |
| Minor comment Table 1b reports means for T1, T4 and T5. Was data from Time 1 included in the study or is this a typo? | We thank the reviewer for pointing out this typo, and adjusted it as follows (page 9):  M(SD) range/percentage per measurement (T3, T4, and T5) |
|  |  |
| Comments from Reviewer 2:   The present paper focused on a subsample of children and young people (CYP) with anxiety disorders, examined the prospective association between behavioral problems and anxiety at 6-year follow-up, and the proposed mediation by parental rejection. The study also tested the relationship between reward or punishment sensitivity and behavioral problems. The authors should be commended for the longitudinal design using a database, and considering parenting rejection as an important contextual aspect for adolescent development. However, there are several major concerns that diminish my enthusiasm for the current study. | We are happy to read that the reviewer indicates the strengths of our study, including the longitudinal design and considering parental rejection as a contextual aspect for adolescent development.  The reviewer also indicated several concerns with our study. We adjusted our study in line with the reviewer’s comments. We did this by including a post-hoc moderation analysis and by elaborating in our discussion on our significant findings and an alternative explanation for why people with high reward sensitivity display more aggressive or disruptive behavior. Below, you will find a more detailed description of our adjustments. |
| P4, “However, given their high reward expectancy, they are also more prone to detect non-reward (rewards with a lower than expected frequency or lower level of reward), which may lead to anger and oppositional behaviors out of frustrative non-reward.” Besides this logic, there is also another possibility that people with high reward sensitivity display more aggressive or disruptive behavior to get the reward when being hindered. | We thank the reviewer for offering this interesting additional view on why people with high reward sensitivity display more aggressive or disruptive behavior. This alternative explanation (in bold) was added in our discussion in the following way (page 34):  “Self-report measures are well-suited to test the responsivity and motivation components of punishment and reward sensitivity. Multiple studies have found associations between self-report measures of punishment sensitivity with anxiety symptoms/disorders in CYP and adults (Bijttebier et al., 2009; Vervoort et al., 2010) and between self-report measures of reward sensitivity and behavioral problems in CYP and adults (Bijttebier et al., 2009; Harmon-Jones, 2003; Morgan et al., 2014; Smits & Kuppens, 2005). Most of these previous studies were cross-sectional and did not focus on comorbid behavioral problems in highly anxious CYP. We do therefore not yet know whether these associations also exist prospectively and whether self-reported punishment and reward sensitivity are related to comorbid behavioral problems in anxious CYP. **Additionally, the current study did not take into account that people with high reward sensitivity may not only display anger out of frustrative non-reward, but may also display more aggressive or disruptive behavior to get the reward when being hindered. Future research that differentiates between these two types of situations is necessary to disentangle these two different explanations. One option would be to employ a scenario approach with scenarios that refer to non-reward situations as well as situations in which the reward could still be gained, and assess participants’ emotions and behaviors in these situations as a function of their reward-sensitivity (e.g., Kreuze et al., 2021).** |
| P5, for the third aim of the current study, I think that parental rejection as a typically harmful environment for adolescents is better to considered as a moderator rather than mediator for the relation between problem behavior in adolescents and later anxiety symptoms in young adulthood. Rather, reward or punishment bias can be served as both moderator or mediator in this relation. I suggest authors run this model in the data analyses. | In line with the reviewer’s comment, we conducted a simple moderation analysis (using Hayes model 1) with behavioral problems in adolescence as independent variable, anxiety in adolescence as covariate, anxiety in young adulthood as dependent variable and parental rejection as moderator. As can be seen in the results, no significant moderation effect was found. This indicates that parental rejection was neither a mediator nor moderator in this association. We therefore did not fit a more complex model including the proneness to attend to (non)punishment/(non)reward to the data. Furthermore, in this simple moderation analysis, behavioral problems in adolescence no longer predicted anxiety in young adulthood (p reaching borderline significance for this main effect in the analysis with the moderator included). This effect may be relatively small and the robustness of this main effect needs to bested in future research The results of this moderation analysis and the conclusions are presented as follows: (pages: 28-29) **Post-hoc analysis: moderation analysis** Post-hoc As a typical harmful environment for adolescents, parental rejection could also be considered as a moderator in the relation between behavioral problems in adolescents and later anxiety symptoms in young adulthood. We therefore conducted a simple moderation analysis using Hayes model 1 with behavioral problems in adolescence as independent variable, anxiety in young adulthood as dependent variable and parental rejection as moderator. Anxiety in adolescence was included as covariate. Table 9 *Moderation analysis for the association between behavioral problems in adolescence and anxiety in young adulthood, moderated by parental rejection* \| **Anxiety in young adulthood** \| ***B*** \| ***SE*** \| ***t*** \| ***p*** \| ***95% CI***; **Multiple Regression Model^1^** \| \| \| \| \| \|; ***R^2­^= .240 F (2,555) =* 44.45 p<.001*** \| *Constant* \| .069 \| .085 \| 0.81 \| .420* \| -0.10; 0.24; \| *Behavioral problems* \| .382 \| .211 \| 1.81 \| .071 \| -0.03; 0.80; \| *Parental rejection* \| .083 \| .059 \| 1.40 \| .16 \| -0.03; 0.20; \| *Moderation effect behavioral problems* parental rejection* \| -.169 \| .134 \| -1.26 \| .207 \| -0.43; 0.09; \| *Anxiety in adolescence* \| *.550* \| *.047* \| *11.74* \| *<0.001** \| *0.46; 0.64*; **BP = Behavioral problems in adolescence** **Anx = anxiety in young adulthood** **PR = parental rejection** **Boot = bootstrap** **^1^model includes anxiety in adolescence as covariate** ***significant with α of .05** \| \| \| \| \| \| As can be seen in table 9, no significant moderation effect was found. Therefore, we did not fit a more complex model including punishment - or reward sensitivity. In the discussion the results of this post-hoc moderation analysis (in bold) were included as follows (page 36 & 37): *Association between behavioral problems in adolescence and anxiety symptoms in young adulthood and moderation/mediation by parental rejection* A second aim of this study was to investigate whether behavioral problems in adolescence increased the risk for having higher anxiety symptoms in young adulthood, whether this association was mediated by parental rejection, and whether this was especially the case for people with high punishment sensitivity. **We did not find evidence for the hypothesis that the association between behavioral problems in adolescence and anxiety in young adulthood would be (partially) mediated or moderated by parental rejection.** This is inconsistent with previous findings among high school pupils showing that the prospective relationship between behavioral problems and internalizing symptoms was mediated by both parental rejection and peer rejection (58). One explanation for this apparent discrepancy in findings might be that parental rejection is especially relevant in early adolescence (as in the study of 58) whereas in young adulthood (as in our study) the influence of parents tends to decrease and peer influence is more crucial (59, 65). We expected that punishment sensitivity would moderate the association between parental rejection and anxiety in young adulthood, however, we did not find evidence for this association. This might also be explained by the decreased influence of parents in young adulthood. In the mediation analysis (where parental rejection was included as mediator) we did find a significant main effect of behavioral problems in adolescence predicting a further increase of anxiety symptoms in young adulthood. **However, in the post-hoc moderation analysis (where parental rejection was included as moderator) this main effect of behavioral problems on anxiety symptoms in young adulthood was no longer significant. Therefore, this main effect may be relatively small and future research is needed to assess the robustness of this effect.** Furthermore, the abstract was adjusted as follows (in bold, page 1): “….We also investigated the prospective association between behavioral problems and anxiety at 6-year follow-up, and the proposed mediation by parental rejection. PS and RS showed no cross-sectional or prospective relationships with comorbid behavioral problems in highly anxious CYP. Yet, behavioral problems in adolescence showed a small prospective relationship with anxiety in young adulthood, **but this was not mediated nor moderated by parental rejection.** No evidence was found for PS/RS being involved in comorbid behavioral problems in anxious CYP. Findings point to comorbid behavioral problems as potential factor contributing to the further increase of anxiety.” |
| P22, authors should give more explanation for the significant correlation results.  In short, it is acceptable that the main hypotheses were not found in the current findings. However, authors should have a clear logic about the main variables and give more deep discussion about the current findings.  Kind regards, | We agree with the reviewer that some more information on the significant correlation results would be helpful. We therefore adapted the discussion as follows:  (page 32 & 33)  “People with high punishment sensitivity are expected to be more inclined to experience threat, and were, therefore, also expected to be more inclined to respond with reactive aggression/anger in threat situations where they cannot avoid (18–20). We thus expected punishment sensitivity to be a risk factor for developing comorbid behavioral problems in anxiety disordered CYP. In line with this hypothesis, we did find a significant positive association between attentional proneness for punishing cues with short cue delay and behavioral problems in young adulthood. This might indicate that an inclination to experience threat in adolescence increases the chance of showing behavioral problems (which are expected to result as defensive response to threat) in young adulthood. However, the Bayesian analyses showed that the strength of the evidence for this finding should be considered inconclusive. Additionally, we would expect this association between an attentional proneness for punishing cues with short cue delay and behavioral problems to also arise cross-sectionally, however, this was not the case. Furthermore, we neither found the expected association between an attentional proneness to non-punishing cues and behavioral problems in adolescence nor in young adulthood. Therefore, the findings provided no clear evidence in support of our hypothesis.”  (page 35& 36)  “We also expected that people with high reward sensitivity would be more prone to detect potential rewards in the environment, and would therefore also be more prone to detect non-reward, which might lead to comorbid behavioral problems out of frustrative non-reward (20,22,24,27,28). In line with our expectation, we did find a significant cross-sectional association between the proneness for rewarding cues with long cue delay and behavioral problems in anxious adolescents. However, the Bayesian analyses showed that the strength of the evidence for this finding should be considered inconclusive. Additionally, we would expect this association to also be present prospectively, however, this was not the case. Also, we neither found the expected association between an attentional proneness for rewarding cues with short cue delay and behavioral problems in adolescence, nor in young adulthood. Therefore, the findings provided no clear evidence that attentional proneness to reward cues was a risk factor for comorbid behavioral problems in highly anxious CYP.”  Nevertheless, in general, the data did not support our main hypotheses.  We had clear predictions with regard to the associations between attentional proneness to (non)punishing cues/rewarding cues and behavioral problems in a highly anxious sample. Only two of the predicted associations were confirmed and the Bayesian analyses indicated that the evidence for these findings was inconclusive. Also, false positive finding cannot be ruled out. The current sample was highly suitable for answering our research questions, given that it was taken from a large prospective cohort study with relatively low dropout and low sampling bias. Taken together, we conclude that reward and punishment sensitivity as indexed by heightened attentional proneness to general cues of punishment and/or reward do not seem to be risk factors for the development of comorbid behavioral problems in highly anxious CYP. |

Furthermore, in addition to the responses to the reviewers, additional points needed to be addressed and were addressed as follows:

| 1. Please ensure that your manuscript meets PLOS ONE's style requirements, including those for file naming. The PLOS ONE style templates can be found at  <https://journals.plos.org/plosone/s/file?id=wjVg/PLOSOne_formatting_sample_main_body.pdf> and  <https://journals.plos.org/plosone/s/file?id=ba62/PLOSOne_formatting_sample_title_authors_affiliations.pdf> | We adjusted the manuscript to meet PLOS ONE’s style requirements, which included adjusting the headers and the reference style. |
| --- | --- |
| 2. Please ensure that you include a title page within your main document. We do appreciate that you have a title page document uploaded as a separate file, however, as per our author guidelines (<http://journals.plos.org/plosone/s/submission-guidelines#loc-title-page>) we do require this to be part of the manuscript file itself and not uploaded separately.  Could you therefore please include the title page into the beginning of your manuscript file itself, listing all authors and affiliations. | The title page was now included in the main document. |
| We note that the grant information you provided in the ‘Funding Information’ and ‘Financial Disclosure’ sections do not match.  When you resubmit, please ensure that you provide the correct grant numbers for the awards you received for your study in the ‘Funding Information’ section. | This is the right funding information that should be included when the study is published. Not all matching codes could be found in the submission program.  This study was funded by the PhD fund awarded to Leonie Kreuze (the first author) by the faculty of Behavioural and Social sciences at the University of Groningen, the Netherlands. This research is part of the TRacking Adolescents’ Individual Lives Survey (TRAILS). Organizations participating in TRAILS include various departments of the University Medical Center and University of Groningen, the Erasmus University Medical Center Rotterdam, Utrecht University, the Radboud Medical Center Nijmegen, and the Parnassia Bavo group, all in the Netherlands. TRAILS has been financially supported by various grants from NWO, ZonMW, GB-MaGW, the Dutch Ministry of Justice, the European Science Foundation, BBMRI-NL, and the participating universities. The funders had no role in study design, data collection and analysis, decision to publish, or preparation of the manuscript. |
| 4. We note that you have indicated that data from this study are available upon request. PLOS only allows data to be available upon request if there are legal or ethical restrictions on sharing data publicly. For more information on unacceptable data access restrictions, please see <http://journals.plos.org/plosone/s/data-availability#loc-unacceptable-data-access-restrictions>.  In your revised cover letter, please address the following prompts:  a) If there are ethical or legal restrictions on sharing a de-identified data set, please explain them in detail (e.g., data contain potentially sensitive information, data are owned by a third-party organization, etc.) and who has imposed them (e.g., an ethics committee). Please also provide contact information for a data access committee, ethics committee, or other institutional body to which data requests may be sent.  b) If there are no restrictions, please upload the minimal anonymized data set necessary to replicate your study findings as either Supporting Information files or to a stable, public repository and provide us with the relevant URLs, DOIs, or accession numbers. For a list of acceptable repositories, please see <http://journals.plos.org/plosone/s/data-availability#loc-recommended-repositories>.  We will update your Data Availability statement on your behalf to reflect the information you provide. | We included this information in our revised cover letter as follows:  **With regard to funding, the following situation holds:**   a) If there are ethical or legal restrictions on sharing a de-identified data set, please explain them in detail (e.g., data contain potentially sensitive information, data are owned by a third-party organization, etc.) and who has imposed them (e.g., an ethics committee). Please also provide contact information for a data access committee, ethics committee, or other institutional body to which data requests may be sent.   Here you find the explanation for our data sharing restrictions and information on data access:  **Data Availability:** Under the General Data Protection Regulation (GDPR), our dataset is considered pseudonymized rather than anonymized, and is still regarded as personal data. When participants were invited to the cohort more than 20 years ago, they were not asked to give informed consent to make their personal data publicly available in pseudonymized form. As a result of this, legal and ethical restrictions prevent the authors from making data from the TRAILS Study publicly available. Data and syntax are available upon request from the TRAILS data manager ([trails@umcg.nl](mailto:trails@umcg.nl)). Detailed information about the participation agreements with TRAILS participants is available from the ethics committee; Central Committee on Research Involving Human subjects (CCMO; [tc@ccmo.nl](mailto:tc@ccmo.nl)). For more information about accessing data from the TRAILS Study, please see <https://www.trails.nl/en/hoofdmenu/data/data-use>. |
| 5. Please upload a copy of Figure S1, to which you refer in your text on page 12. If the figure is no longer to be included as part of the submission please remove all reference to it within the text. | Figure S1 was uploaded. |
| 6. Please include captions for your Supporting Information files at the end of your manuscript, and update any in-text citations to match accordingly. Please see our Supporting Information guidelines for more information: <http://journals.plos.org/plosone/s/supporting-information>. | A caption was added for our supporting information including Figure S1. |
| 7. We noticed you have some minor occurrence of overlapping text with the following previous publication(s), which needs to be addressed:  - <https://pubmed.ncbi.nlm.nih.gov/32445103/>  In your revision ensure you cite all your sources (including your own works), and quote or rephrase any duplicated text outside the methods section. Further consideration is dependent on these concerns being addressed. | We adjusted to occurrences of minor overlap and indicate for the method section that this method is similar as the method described in our previous study. |
